# Salt-Adapted Microorganisms: A Promising Resource for Novel Anti-Cancer Drug Discovery

**DOI:** 10.3390/md23080296

**Published:** 2025-07-24

**Authors:** Longteng Fang, Liping Xu, Marhaba Kader, Tingting Ding, Shiyang Lu, Dong Wang, Amit Raj Sharma, Zhiwei Zhang

**Affiliations:** 1 School of Pharmaceutical Sciences and Institute of Materia Medica, Xinjiang University, Urumqi 830017, China; 107552403438@stu.xju.edu.cn (L.F.); 107552303680@stu.xju.edu.cn (L.X.); 107552203666@stu.xju.edu.cn (M.K.); 20231106007@stu.xju.edu.cn (T.D.); 2 School of Life Science and Technology, Xinjiang University, Urumqi 830017, China; 3 Analysis and Testing Center, Shenyang University of Chemical Technology, Shenyang 110142, China; lushiyang@foxmail.com; 4 Arkansas Biosciences Institute, Arkansas State University, Jonesboro, AR 72401, USA

**Keywords:** salt-adapted microorganisms, halophilic and halotolerant microorganisms, secondary metabolites, anticancer activity, microbial resources

## Abstract

Microorganisms serve as a vital source of natural anticancer agents, with many of their secondary metabolites already employed in clinical oncology. In recent years, salt-adapted microbes, including halophilic and halotolerant species from marine, salt lake, and other high-salinity environments, have gained significant attention. Their unique adaptation mechanisms and diverse secondary metabolites offer promising potential for novel anticancer drug discovery. This review consolidated two decades of research alongside current global cancer statistics to evaluate the therapeutic potential of salt-adapted microorganisms. Halophilic and halotolerant species demonstrate significant promise, with their bioactive metabolites exhibiting potent inhibitory effects against major cancer cell lines, particularly in lung and breast cancer. Evidence reveals structurally unique secondary metabolites displaying enhanced cytotoxicity compared to conventional anticancer drugs. Collectively, salt-adapted microorganisms represent an underexplored yet high-value resource for novel anticancer agents, offering potential solutions to chemotherapy resistance and treatment-related toxicity.

## 1. Introduction

Cancer poses a profound threat to human health and has consistently remained a central challenge in medical research and clinical practice throughout history. The current global situation for cancer prevention and treatment is increasingly severe. Projections suggest that by 2050, the annual number of new cancer cases worldwide will reach 35 million, a 77% increase from 2022 [1]. Cytotoxic natural products continue to be a core source of chemotherapeutic agents [2]. However, the evolution of cancer resistance and therapeutic side effects limits their clinical application. Therefore, there is an urgent need to explore novel anticancer chemotherapeutic agents to overcome the limitations of existing treatments. In this context, the investigation into the extraction of cytotoxic substances from salt-adapted microorganisms has attracted significant scholarly attention [3]. Salt-adapted microorganisms are defined as microbial groups capable of surviving and reproducing normally in salt-containing habitats, which are primarily classified into halophilic and halotolerant microorganisms. These microorganisms can thrive in diverse environments with varying salt concentrations, including salt lakes, deep-sea regions, saline soils, marshes, deep-sea sediments, and anthropogenic hypersaline environments; all such habitats harbor abundant communities of halophilic and halotolerant microorganisms [4]. Special ecological factors, such as high salt concentration and low water activity, have led these microorganisms to develop diverse metabolic types, including aerobic and anaerobic phototrophs, aerobic chemotrophic organic heterotrophs, and chemolithotrophic autotrophs [5]. Through long-term adaptation to saline environments and natural selection, halophilic and halotolerant microorganisms have gradually evolved unique physiological structures, functions, and genetic mechanisms, resulting in significant metabolic diversity. This diversity enables them to produce various metabolites with unique structures and functions, some of which have demonstrated significant cytotoxic and antimicrobial effects [6,7]. Therefore, halophilic and halotolerant microorganisms have become a promising resource for drug discovery and development. To date, research and applications using these microorganisms have yielded numerous positive results in the medical field.

Among the various studies on halophilic and halotolerant microorganisms, many researchers have focused on cancer therapy and antimicrobial research, aiming to explore the potential of their metabolites in anticancer and antimicrobial applications. These studies not only provide new ideas and methods for cancer treatment but also offer effective drug resources for inhibiting pathogenic microorganisms. This review systematically summarizes the cytotoxic effects of bioactive metabolites from halophilic and halotolerant microorganisms against tumors, based on two decades of research literature and experimental data. It focuses on exploring their therapeutic potential in globally high-incidence cancers, aiming to strengthen the theoretical basis of these microorganisms in anticancer research and provide insights for novel drug development.

## 2. Halophilic/Halotolerant Microorganisms

Halophilic microorganisms thrive in environments with a minimum salinity of 0.2 M NaCl (1.17% salt concentration), whereas halotolerant microorganisms can survive and proliferate across a broad range of salinities, including high-salt conditions [8]. These microbes have evolved specialized physiological and metabolic adaptations to withstand osmotic stress, ion toxicity, and other challenges posed by saline habitats. Due to their remarkable resilience, halophiles and halotolerant species are key contributors to high-salinity ecosystems worldwide. They participate in intricate ecological networks, driving nutrient cycling, energy transfer, and ecosystem stability. Their interactions with the environment and other organisms make them essential for maintaining biodiversity and ecological balance in extreme habitats [9,10,11,12].

Earth hosts a rich diversity of halophilic and halotolerant microorganisms, with marine environments serving as one of their primary habitats. Approximately 70% of the Earth’s surface is covered by seawater, with an average salinity ranging between 33 and 37 grams per liter [13]. In addition to marine habitats, inland saline lakes such as the Dead Sea, Great Salt Lake, Lake Van, Lake Urmia, and others are abundant in these microorganisms [14]. Moreover, saline soils also harbor a diverse array of halophilic and halotolerant species. Alkaline and saline soils cover approximately 93.2 million hectares globally, providing unique niches for these microbes [15]. Notably, these microorganisms also inhabit human-impacted environments, including saline industrial effluents, wastewater, and fermented foods [16].

The mechanisms by which halophilic and halotolerant microorganisms adapt to saline environments are undergoing research, but can generally be categorized into two primary strategies. The first involves the active accumulation of K^+^ and Cl^−^ ions within the cell when exposed to high salinity, coupled with specific ion transport mechanisms that expel Na^+^ ions, thereby maintaining osmotic balance and stabilizing internal salt concentrations to match the external saline conditions [17,18,19]. The second strategy focuses on maintaining low intracellular salt concentrations by synthesizing and accumulating compatible solutes—low-molecular-weight, water-soluble, organic compounds such as tetrahydropyrimidine, betaine, trehalose, glutamate, and proline. These solutes play vital roles in enhancing the cell’s adaptability to osmotic stress, stabilizing cellular structures, and preserving the functionality of intracellular macromolecules [20,21,22]. Additionally, the remarkable survival capabilities of these microorganisms in high-salinity environments are closely linked to their unique proteins, enzymes, and cell membrane structures, which have evolved specific physicochemical properties and functional mechanisms to thrive under extreme conditions [23,24,25].

## 3. Cytotoxic Secondary Metabolites from Halophilic and Halotolerant Microorganisms

Natural products represent an invaluable resource for anticancer drug discovery, with approximately 25% of anticancer drugs approved worldwide from 1981 to 2019 being directly or indirectly derived from natural sources [26]. Among these, microbial metabolites have emerged as particularly promising candidates due to their exceptional metabolic diversity, favorable biocompatibility, low immunogenicity, and scalability for industrial production [27]. In recent years, extremophilic microorganisms, particularly halophilic and halotolerant species, have attracted considerable research interest due to their unique metabolic pathways and bioactive secondary metabolites. These microorganisms produce compounds that exhibit potent cytotoxicity against various cancer cell lines, with some showing superior anticancer efficacy compared to first-line clinical drugs, such as paclitaxel and cisplatin [28,29]. Anticancer agents derived from halophilic microbes often have novel mechanisms of action and reduced toxicity profiles, offering distinct advantages over conventional chemotherapeutic drugs. Their ability to thrive in extreme environments suggests an untapped potential for structurally unique and biologically active molecules, positioning them as a precious frontier in anticancer drug development.

### 3.1. Anti-Lung Cancer

Lung cancer remains the most lethal malignancy worldwide, characterized by high incidence rates and complex pathological features that present significant therapeutic challenges. Despite advances in targeted therapy and immunotherapy, critical limitations persist, including drug resistance, variable response rates, toxicity, and accessibility issues [30,31]. In recent years, halophilic and halotolerant microorganisms have emerged as a promising source of bioactive metabolites with unique structural and functional properties. Remarkably, studies have found that these metabolites exhibit potent inhibitory effects on multiple lung cancer cell lines, including the human lung adenocarcinoma cell line A549, human large cell lung cancer cell line NCI-H460, human lung adenocarcinoma cell line H1975, human lung cancer cell line H2887, and human lung adenocarcinoma cell line Calu-3, highlighting their potential as novel anticancer agents. This section presents a systematic evaluation of 23 halophilic/halotolerant microbial species and 55 of their metabolites with demonstrated anti-lung cancer activity. Systematically categorizing these compounds by microbial origin and structural class may elucidate their therapeutic potential, offering novel avenues for innovative lung cancer treatment strategies.

#### 3.1.1. Actinomycetes

**Figure 1:**
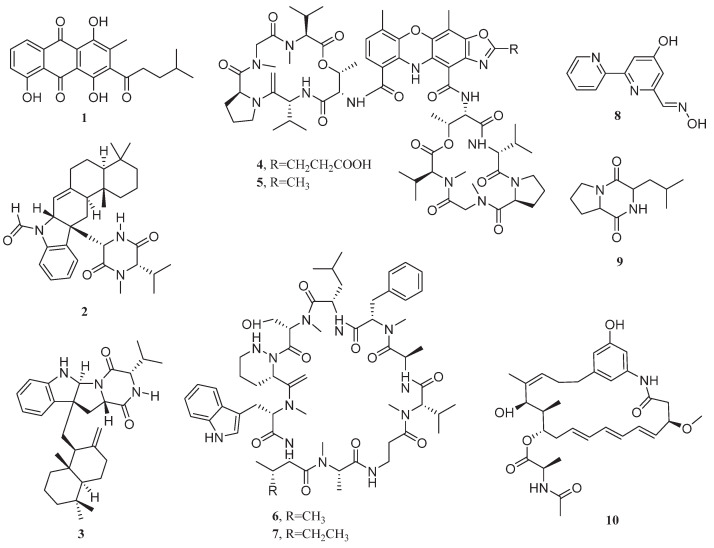
Chemical structures of compounds **1**–**10**.

In the exploration of anti-lung cancer active substances from halophilic and halotolerant microorganisms, actinobacteria dominate due to their unique secondary metabolism potential. Within this phylum, the genus *Streptomyces* plays a central role, producing a variety of bioactive compounds that exhibit significant inhibitory effects on lung cancer cells. Moreover, the chemical structure of the most promising novel natural products is shown in Figure 1.

In 2012, the anthraquinone derivative galvaquinone B (**1**) was isolated from *Streptomyces spinoverrucosus* SNB-032, an actinomycete derived from marine sediments. This compound displayed cytotoxic effects on H2887 and Calu-3 cells, with IC_50_ values of 5.0 and 12.2 μM, respectively. The C-1 hydroxyl group in its molecular structure was confirmed as a critical functional moiety for bioactivity. Further studies showed that the biosynthesis of this compound relied on a type II polyketide synthase (PKS) gene cluster, and a Baeyer−Villiger-type oxidation rearrangement catalyzed by luciferase-like monooxygenases (RsdO1, RsdO6) and a flavin reductase (RsdO2) served as the key step driving the formation of its skeleton [32,33]. Additionally, two hybrid isoprenoids, indotertine B (**2**) and drimentine G (**3**), were isolated from *Streptomyces* sp. CHQ-64. Compound **3** demonstrated potent cytotoxicity against A549 human lung cancer cells (IC_50_ = 1.01 μM), while compound **2** exhibited moderate activity (IC_50_ = 4.88 μM) [34,35]. Drimentine G has now been artificially synthesized with a total yield of up to 43% [36].

Two new cyclopeptide compounds, neo-actinomycin A (**4**) and neo-actinomycin B (**5**), were reported from the marine sediment-derived *Streptomyces* sp. IMB094, which demonstrated cytotoxicity against A549 cells with submicromolar IC_50_ values of 0.0658 and 0.9523 μM. The C-2 carboxyethyl substituent in neo-actinomycin A significantly enhanced cytotoxic activity compared to the methyl substituent in neo-actinomycin B. This difference may be attributed to the additional hydrogen bond interaction formed by the carboxyl group of the carboxyethyl moiety with DNA, thereby strengthening the compound’s binding to the target [37]. Similarly, two cyclic decapeptides, lenziamide D1 (**6**) and lenziamide B1 (**7**), were isolated from *Streptomyces* sp. S063. These compounds displayed growth inhibitory effects against human cancer cell lines HEL, H1975, H1299, and A549–taxol, with IC_50_ values ranging from 8 to 24 μM. Among them, compound **7** showed significant cytotoxicity toward the H1975, achieving an IC_50_ of 8 μM. Biosynthetic studies indicated that their synthesis relied on a non-ribosomal peptide synthetase (NRPS) gene cluster len containing piperazic acid biosynthesis genes (*lenE/lenF*), involving N-methyltransferase-catalyzed methylation modifications and precursor-directed biosynthetic pathways [38].

In addition to members of the genus *Streptomyces*, secondary metabolites produced by rare actinomycetes from saline environments also exhibit unexpected anti-lung cancer activities. In 2011, one new bipyridine alkaloid, caerulomycin H (**8**), was isolated from the marine actinomycete *Actinoalloteichus cyanogriseus* WH1-2216-6 and exhibited cytotoxicity against A549 cells with an IC_50_ value of 8.4 μM. The compound might be formed by the condensation of lysine-derived picolinic acid with acetyl-CoA, followed by hydroxylation and oximation [39].

#### 3.1.2. Bacteria

The deep-sea sediment-derived bacterium *Staphylococcus* sp. MB30 produced a pyrrolopyrazinedione derivative, pyrrole [1,2-a]pyrazine-1,4-dione, hexahydro-3- (2-methylpropyl) (**9**), which exhibited anti-proliferative activity against A549 lung cancer cells (IC_50_ = 19.94 µg/mL). Mechanistic studies have demonstrated that compound **9** induces G1-phase cell cycle arrest and triggers apoptosis via modulation of Bcl-2 family proteins, which downregulate anti-apoptotic Bcl-2 and Bcl-xL while upregulating pro-apoptotic Bax. This was accompanied by caspase-3 activation and PARP cleavage, collectively driving programmed cell death [40].

From the deep-sea-derived bacterium *Ochrobactrum* sp. OUCMDZ-2164, a novel ansamycin compound, trienomycin H (**10**), was isolated. Trienomycin H demonstrated selective anti-proliferative activity against the A549 cells, with an IC_50_ value of 15 μM [41].

#### 3.1.3. Fungi

**Figure 2:**
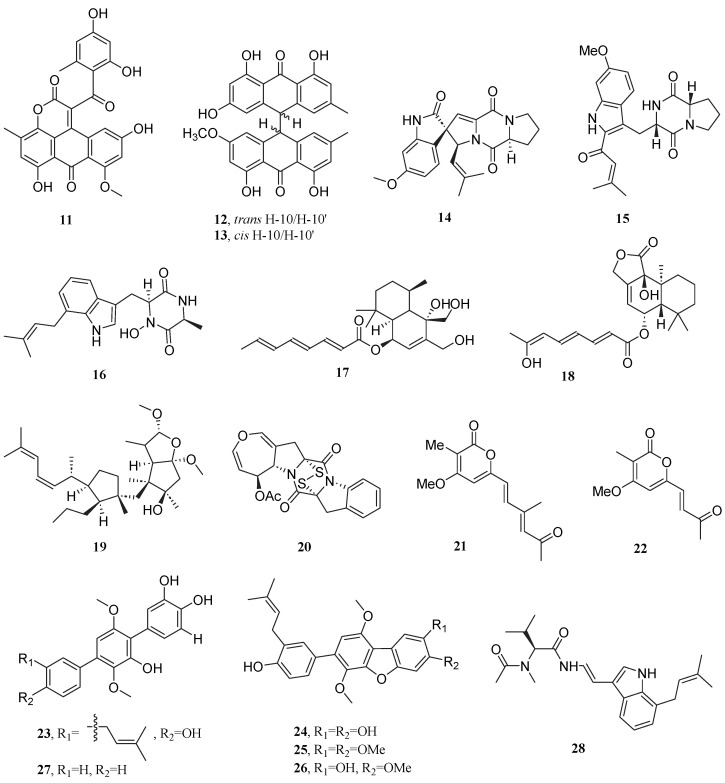
Chemical structures of compounds **11**–**28**.

**Figure 3:**
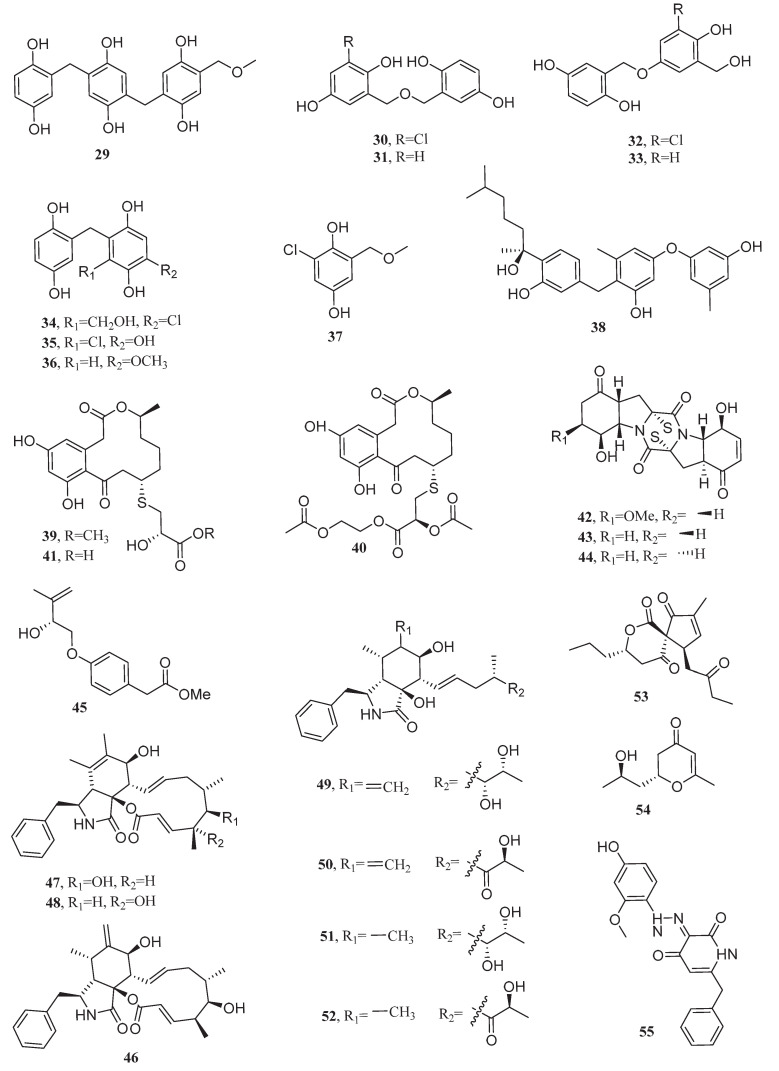
Chemical structures of compounds **29**–**55**.

In microbial drug development systems, eukaryotic microorganisms exhibit more complex secondary metabolic networks compared to prokaryotic microorganisms. These organisms, through highly evolved biosynthetic gene clusters, are capable of producing bioactive molecules with a structural diversity index significantly higher than that of bacterial taxa [42]. Importantly, the majority of fungi discussed subsequently are members of *Ascomycota*, a phylum distinguished by its highly specialized sexual reproduction (via ascospore-forming asci) and remarkable diversity of bioactive secondary metabolites (Figure 2 and Figure 3).

In 2008, an aspergiolide B (**11**) and two bianthrones, (*trans/cis*)-emodin-physcion bianthrones (**12** and **13**) were isolated from the marine-derived fungus *Aspergillus glaucus*. These compounds exhibited potent cytotoxic effects against A549 lung cancer cells, with IC_50_ values of 0.24, 9.20, and 14.20 μM, respectively [43]. The activity of aspergiolide B was potentially related to the C-8 methoxy group and the integrity of the core skeleton. In silico studies based on molecular docking speculated that it might target the EGFR tyrosine kinase, but experimental validation was lacking [44,45]. Meanwhile, *trans*-emodin-physcion bianthrones showed significantly higher activity than the cis-isomers, indicating that the trans-configuration facilitated target binding due to reduced steric hindrance. The distribution of methoxy and phenolic hydroxyl groups influenced their activity. Subsequent studies confirmed that these two compounds were regulated by the same polyketide synthase (PKS) gene cluster and belonged to the emodin/physcion biosynthetic pathway products [46,47]. During the same period, three new diketopiperazine alkaloids, 6-methoxyspirotryprostatin B (**14**), 18-oxotryprostatin A (**15**), and 14-hydroxyterezine D (**16**), produced by the fungus *Aspergillus sydowi*, displayed cytotoxicity against A549 cells with IC_50_ values of 8.29, 1.28, and 7.31 μM, respectively [48].

From the rhizospheric soil of the mangrove plant *Bruguiera gymnorrhiza*, the fungus *Aspergillus ustus* 094102 was isolated. This fungus produced two new drimane-type sesquiterpenes, ustusolate A (**17**) and ustusolate C (**18**), along with a new ophiobolin, 21-*epi*-ophiobolin O (**19**), under approximately 3.3% salt conditions. These three compounds demonstrated potent anti-proliferative activity against A549 cells, with IC_50_ values of 30.00, 10.50, and 0.60 μM, respectively. Interestingly, compound **19** showed comparable efficacy to the clinical anticancer drug etoposide (IC_50_ = 0.63 μM) [49,50].

The marine-derived fungus *Aspergillus* sp. KMD 901, isolated from sediment samples, produced the novel diketopiperazine disulfide deoxyapoaranotin (**20**), which exhibited an IC_50_ value of 23 μM against A549 cells [51]. The endophytic fungus *Aspergillus niger* MA-132, isolated from mangrove plants, can produce two new α-pyrone derivatives in a salt environment: nigerapyrone D (**21**) and nigerapyrone E (**22**). Compound **22** exhibited an IC_50_ value of 43 μM against A549 cells, demonstrating stronger inhibitory effects than the anticancer drug 5-fluorouracil (IC_50_ = 52 μM). In contrast, compound **21** showed weaker activity against A549 cells, with an IC_50_ value of 81 μM [52].

The mangrove-derived fungus *Aspergillus taichungensis* ZHN-7-07 produced prenylated polyhydroxy-*p*-terphenyl metabolites, including prenylterphenyllin A (**23**), prenylcandidusins A-C (**24**–**26**), and a polyhydroxy-*p*-terphenyl derivative 4′′-dehydro-3-hydroxyterphenyllin (**27**). These compounds inhibited A549 cell proliferation with IC_50_ values of 8.32, 53.2, 8.61, 12.26, and 40.71 μM, respectively [53]. Additionally, the salt pan sediment-derived fungus *Aspergillus sclerotiorum* PT06-1 yielded a novel indole derivative, indole-3-ethenamide (**28**), which exhibited anti-proliferative activity against A549 cells with an IC_50_ value of 3 μM in the SRB assay. Its biosynthesis is speculated to occur via condensation of tryptophan and valine, followed by decarboxylation, dehydrogenation, isoprenylation, and N-acylation/methylation [54].

The marine-derived fungus *Penicillium terrestre* yielded a series of bioactive metabolites, including the trimeric gentisyl alcohol derivative terrestrol A (**29**), dimeric terrestrols B-H (**30**–**36**), and the phenolic compound 2-chloro-6- (methoxymethyl)benzene-1,4-diol (**37**). These secondary metabolites showed potent cytotoxicity against A549 cells, with IC_50_ values ranging from 5.7 to 56.5 μM. Of note, terrestrol G **(34**) emerged as the most potent inhibitor, displaying significant anti-proliferative activity (IC_50_ = 5.7 μM), likely attributed to its chlorine atom enhancing polarity, compact carbon−carbon bond conformation, and inhibition of Src/KDR kinases. Other analogs showed weaker activity due to the absence of chlorine or methoxy substitution [55]. The endophytic fungus *Penicillium expansum* 091006 from a mangrove plant produced a polyphenol compound, expansol B (**38**), which inhibited the proliferation of A549 cells with an IC_50_ value of 1.9 μM [56].

Three new sulfur-containing curvularin derivatives, sumalarins A–C (**39**–**41**), were isolated from *Penicillium sumatrense* MA-92. These compounds displayed extremely significant cytotoxicity against NCI-H460 cells with IC_50_ values of 3.8, 4.6, and 7.0 μM, respectively, which were importantly superior to that of 5-fluorouracil (IC_50_ = 8.5 μM) [57]. This represents the first report of sulfur-containing curvularins, and sulfur substitution or a double bond was essential for the cytotoxic activity. Concurrently, disulfide-bridged diketopiperazine derivatives brocazine A (**42**), B (**43**), and F (**44**) from *Penicillium brocae* MA-231 exhibited potent inhibitory activity against NCI-H460 cell proliferation, with IC_50_ values of 4.9, 4.0, and 0.89 μM, all lower than the clinical drug gefitinib (7.6 μM). The cytotoxicity differences among the three compounds originated from the precise regulation of molecular polarity by substituents: the C-7 methoxy group in brocazine A reduced molecular polarity, brocazine B showed significantly enhanced polarity due to demethoxylation, and the C-5′ hydroxyl group in brocazine F further improved hydrophilicity, optimizing the binding efficiency to the target. Studies indicated that the disulfide bridge served as an essential structure for the cytotoxicity of these compounds, and its absence completely abolished the activity [58].

The hydroxyphenylacetic acid derivative (2′*R*)-westerdijkin A (**45**) isolated from *Penicillium chrysogenum* LD-201810 displayed moderate cytotoxicity against A549 cells with an IC_50_ value of 70.0 μM [59].

The marine-derived fungus *Spicaria elegans* produced the alkaloid compounds cytochalasins Z_7_–Z_9_ (**46**–**48**), which displayed cytotoxicity against A549 cells with IC_50_ values of 8.8, 21, and 8.7 μM. Their cytotoxicity was closely associated with the polarity and unsaturation of the hexacyclic substituents [60,61]. Through subsequent multiple subculture rejuvenation and large-scale fermentation of the same strain, isolation yielded additional cytochalasins Z_10_–Z_13_ (**49**–**52**). These compounds also demonstrated cytotoxic activity against A549 cells, with IC_50_ values of 9.6, 4.3, 92.0, and 76.0 μM, respectively [62].

In 2021, hawatide D (**53**), a highly oxygenated polyketide, was isolated from the deep-sea-derived fungus *Paraconiothyrium hawaiiense* FS482. This structurally complex natural product demonstrated moderate cytotoxic activity against the cell line A549, with an IC_50_ value of 53.34 μM [63].

The halotolerant fungus *Cladosporium halotolerans* FS702 produced the novel pyranone derivative (*R*)-6-((8*R*)-hydroxypropyl)-2-methyl-5,6-dihydro-4H-pyran-4-one (**54**), which exhibited remarkable anti-proliferative activity against A549 cells (IC_50_ = 0.23 μM). Notably, compound **54** demonstrated 6-fold greater potency than the clinical chemotherapeutic agent doxorubicin (IC_50_ = 1.38 μM) in this assay [64]. *Talaromyces amestolkiae* HDN21-0307, isolated from deep-sea cold seep sediments, produced the phenylhydrazone alkaloid talarohydrazone A (**55**), which possessed a rare pyridinedione-phenylhydrazone skeleton and exhibited cytotoxicity against NCI-H446 cells with an IC_50_ value of 4.1 μM [65].

Lung cancer remains the leading cause of cancer-related deaths worldwide. Current treatments are often hindered by drug resistance, toxicity, and inconsistent efficacy. Halophilic and halotolerant microorganisms are known for their unique metabolic pathways and bioactive secondary metabolites, thus representing a promising source of novel anti-cancer agents. This study systematically evaluated 23 microorganisms and 55 bioactive compounds with demonstrated anti-lung cancer activity. These were categorized by microbial source (actinomycetes, bacteria, and fungi) and structural class. Actinomycetes, particularly *Streptomyces* species, dominate this field, producing potent cytotoxic compounds, such as 21-*epi*-ophiobolin O (**19**), exhibiting potent cytotoxicity against A549 cells, with an IC_50_ value of 0.60 μM, comparable to the reference drug etoposide (IC_50_ = 0.63 μM). The observed activity suggests that the 2,5-dimethoxyl-2H,3H, 5H-furan moiety may serve as a critical pharmacophore contributing to its cytotoxic effects. The fungus *Aspergillus taichungensis* produced pyranone derivative nigerapyrone E (**22**), which demonstrated approximately 12-fold greater inhibitory effects against A549 cells than 5-FU. Similarly, the sulfur-containing polyketide derivatives sumalarins A-C (**39**–**41**) demonstrated superior inhibitory activity against NCI-H460 lung cancer cells compared to the standard chemotherapeutic agent 5-FU (Table 1). The structurally novel and significantly cytotoxic metabolites produced by salt-adaptive microorganisms hold promise for bringing transformative innovative opportunities to lung cancer therapy. Their mechanism of action may overcome tumor drug resistance and enhance therapeutic efficacy by targeting novel therapeutic targets.

### 3.2. Anti-Breast Cancer

Breast cancer remains one of the most prevalent malignancies among women worldwide, and optimizing treatment strategies continues to be a critical focus in oncology research. While modern medical interventions have made significant progress, persistent challenges such as drug resistance and treatment-related side effects hinder clinical outcomes. These limitations have driven scientists to investigate unique microbial resources from specialized habitats, including salt environments. Studies have found that secondary metabolites extracted from halophilic (halotolerant) microorganisms exhibit potent cytotoxicity against human breast cancer cell lines MCF-7, MDA-MB-231, and MDA-MB-468. The bioactive compounds produced by these salt-adapted microorganisms represent a promising avenue for developing novel therapies, potentially overcoming current barriers in breast cancer treatment and paving the way for more effective and tolerable therapeutic options.

#### 3.2.1. Actinomycetes

**Figure 4:**
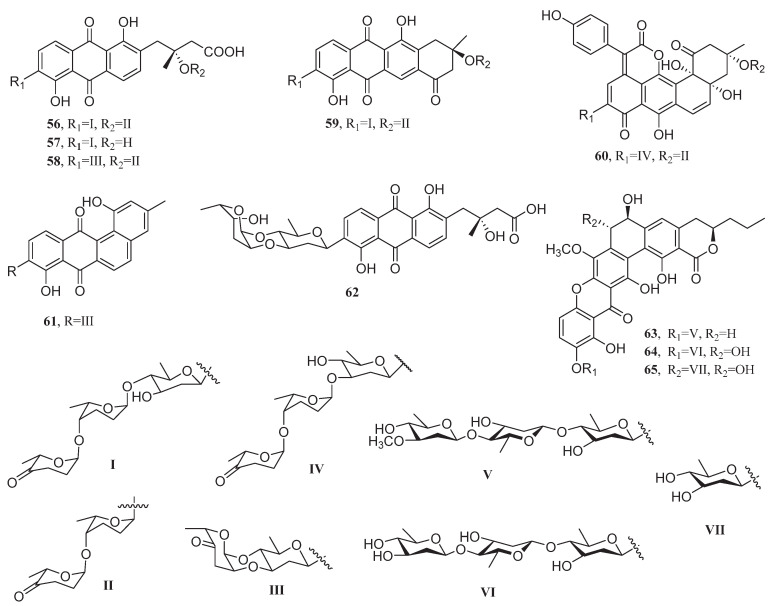
Chemical structures of compounds **56**–**65**.

*Streptomyces lusitanus* SCSIO LR32, a deep-sea actinomycete isolated from South China Sea sediments at a depth of 3,370 m, produced five novel C-glycoside angucyclines grincamycins B–F (**56**–**60**) (Figure 4). These compounds demonstrated remarkable cytotoxicity against the cell line MCF-7, outperforming conventional chemotherapeutics. Grincamycins B–F exhibit IC_50_ values of 12.0, 11.0, 6.1, 8.7, and 19.0 μM, respectively, surpassing the efficacy of both 5-fluorouracil (IC_50_ = 35 μM) and doxorubicin (IC_50_ = 6.9 μM) [66]. Similarly, *Streptomyces* sp. SCSIO 11594, another deep-sea-derived actinomycete, was found to produce the C-glycoside angucycline marangucycline B (**61**), which demonstrated exceptional cytotoxicity against MCF-7 breast cancer cells (IC_50_ = 0.24 μM). This activity significantly surpassed that of the clinical chemotherapeutic agent cisplatin (IC_50_ = 5.26 μM) in the same cell line [67].

The angucycline glycoside vineomycin E (**62**), isolated from the marine bacterium *Streptomyces* sp. OC1610.4 displayed cytotoxicity against three breast cancer cell lines, MCF-7, MDA-MB-231, and BT-474, with IC_50_ values of 6.07, 7.72, and 4.27 μM, respectively [68].

Polycyclic xanthones kebanmycins A–C (**63**–**65**), first isolated from *Streptomyces* sp. SCSIO 40068 (a mangrove rhizosphere sediment-derived actinomycete), exhibited significant cytotoxicity against MCF-7 cells, with IC_50_ values of 0.12, 1.8, and 0.76 μM, respectively. Among them, compound **63** displayed superior cytotoxicity to adriamycin against MCF-7 cells (IC_50_ = 0.72 μM) [69].

**Figure 5:**
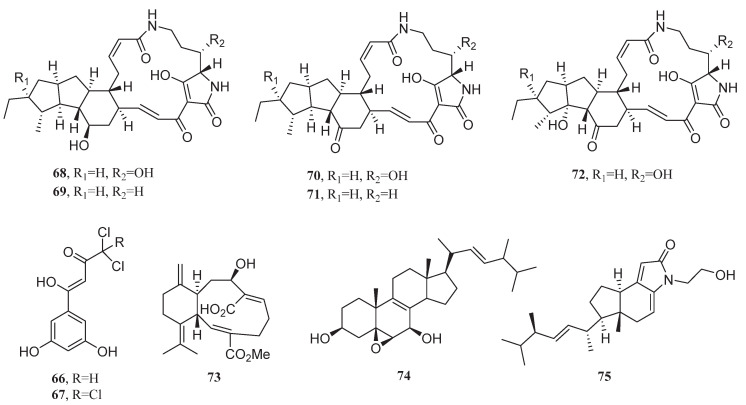
Chemical structures of compounds **66**–**75**.

In 2013, two novel chlorinated polyketides, strepchloritides A–B (**66**–**67**), were isolated from *Streptomyces* sp. OUCMDZ-1703, an endophytic strain derived from soft coral (Figure 5). These compounds displayed significant cytotoxic activity against the human breast cancer cell line MCF-7, with IC_50_ values of 9.9 μM (**66**) and 20.2 μM (**67**), respectively [70]. Their structural uniqueness, featuring chlorine substitution, underscores the potential of marine-derived *Streptomyces* as a source of bioactive polyketides.

Polycyclic tetramate macrolactams 10-*epi*-HSAF (**68**), 10-*epi*-deOH-HSAF (**69**), 10-*epi*-maltophilin (**70**), 10-*epi*-xanthobaccin C (**71**), and 10-*epi*-hydroxymaltophilin (**72**), isolated from *Streptomyces* sp. SCSIO 40010 exhibited significant cytotoxicity against MCF-7 cells, with IC_50_ values ranging from 2.47 to 6.83 μM. Among them, 10-*epi*-HSAF (**68**) demonstrated superior inhibitory effects on MCF-7 cells (IC_50_ = 2.47 μM) compared to cisplatin (IC_50_ = 3.19 μM). Its high activity was attributed to C-7 dehydroxylation reducing molecular polarity to enhance membrane permeability, and the ketone conformation optimizing target binding efficiency [71,72].

Coral endophytic bacterium *Streptomyces albogriseolus* SY67903 produced a eunicellin diterpenoid microeunicellol A (**73**), which inhibited the proliferation of MCF-7 and MDA-MB-231 cells with IC_50_ values of 5.3 μM for MCF-7 cells and 8.6 μM for MDA-MB-231 cells. The C-14/C-18 double bond in the structure of this compound served as the key structural unit for maintaining cytotoxicity, and its absence led to a significant loss of activity [73].

**Figure 6:**
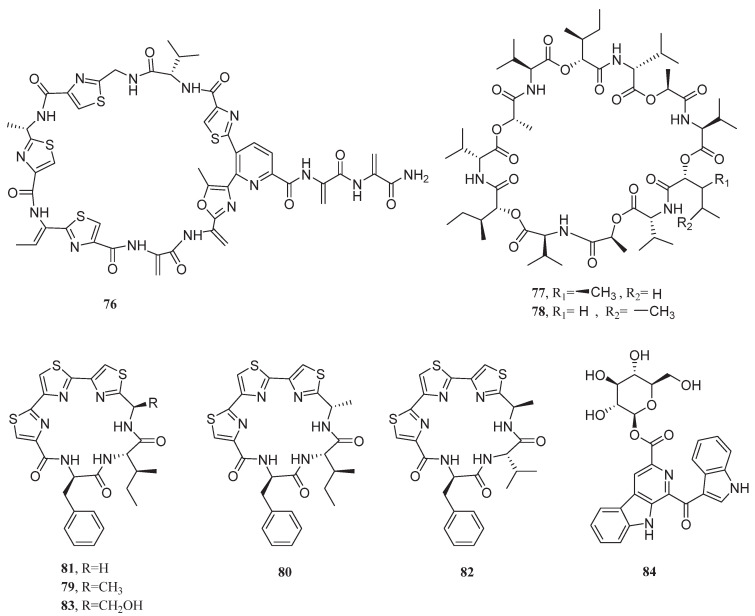
Chemical structure of compounds **76**–**84**.

In 2017, the ergosterol ananstrep C (**74**) and steroidal alkaloid anandin A (**75**) produced by the bacterium *Streptomyces anandii* H41-59 exhibited inhibitory activities against MCF-7 cells with IC_50_ values of 18.1 and 7.5 μg/mL, respectively. The activity of the former was related to its epoxy/double bond structure, while the latter relied on lipophilic optimization mediated by the lactam ring/double bond [74,75]. The halotolerant actinomycete *Streptomonospora* sp. DSM 106425T, isolated from sandy beach sediments, produced a novel thiopeptide antibiotic, litoralimycin A (**76**), which exhibited an IC_50_ value of 1.0 μg/mL against MCF-7 cells. Its cytotoxicity was closely associated with the side-chain dehydroalanine unit (Figure 6) [76].

#### 3.2.2. Bacteria

The endophytic bacterium *Bacillus silvestris* isolated from a marine crab produced two cyclodepsipeptides, bacillistatin 1 (**77**) and bacillistatin 2 (**78**), which were cytotoxic to MCF-7 cells with GI_50_ values of 0.00061 and 0.00031 μg/mL, respectively. Particularly, bacillistatin 2 demonstrated particularly potent inhibitory effects on MCF-7 cells, surpassing valinomycin (GI_50_ = 0.001 μg/mL) [77].

*Bacillus* sp. B19-2 produced cyclic peptides bathiapeptides A_1_ (**79**), A_2_ (**80**), B (**81**), C (**82**), and D (**83**), which exhibited inhibitory activities against MCF-7 cells with the IC_50_ value ranging from 0.5 to 12.4 μM. Especially, bathiapeptide A1 displayed extraordinary cytotoxicity against MCF-7 cells, achieving sub-micromolar potency (IC_50_ = 0.5 μM) that dramatically outperformed conventional cisplatin (IC_50_ = 19.1 μM) by 38-fold [78].

In 2022, a novel glycosylated bisindole alkaloid, pityriacitrin D (**84**), was isolated from the bacterium *Bacillus siamensis* 168CLC-66.1. This compound exhibited moderate inhibitory activity against MDA-MB-231 cells with a GI_50_ value of 8.0 μM [79].

#### 3.2.3. Fungi

**Figure 7:**
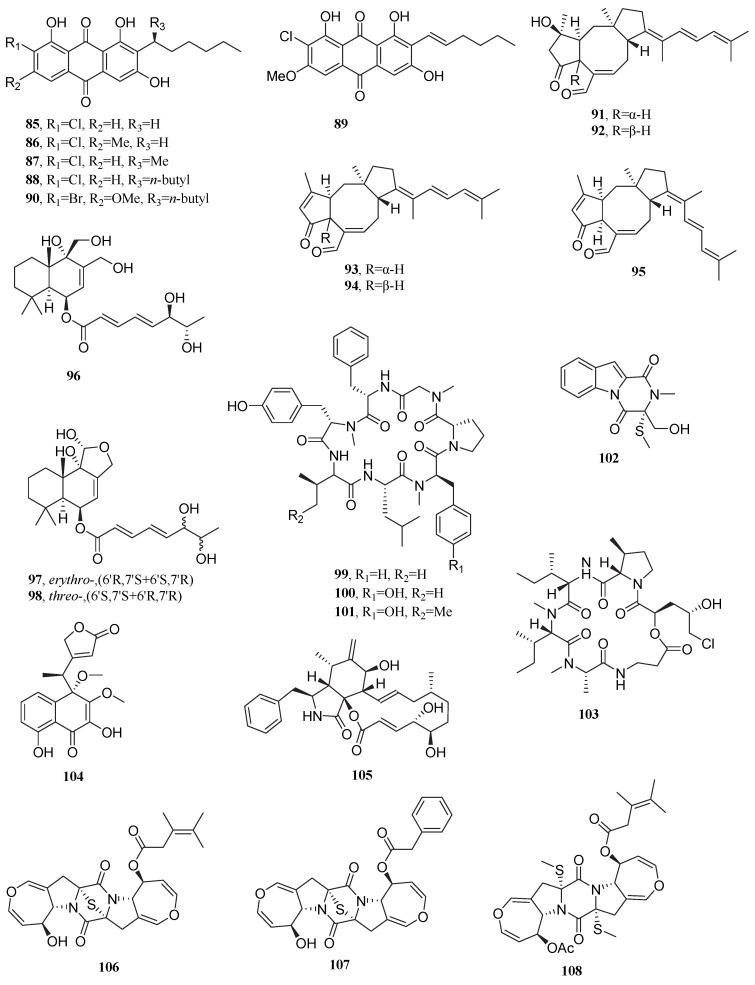
Chemical structures of compounds **85**–**101**.

The chlorinated anthraquinones (1′*S*)-7-chloroaverantin (**85**), (1′*S*)-6-O-methyl-7-chloroaverantin (**86**), (1′*S*)-1′-O-methyl-7-chloroaverant-in (**87**), (1′*S*)-7-chloroaverantin-1′-butyl ether (**88**), 6-O-methyl-7-chloroaver-ythrin (**89**) and (1′*S*)-6-O-Methyl-7-bromoaverantin (**90**) were first isolated from the deep-sea fungus Aspergillus sp. SCSIO F063, which exhibited significant cytotoxicity against MCF-7 cells, with IC_50_ values ranging from 6.64 to 49.53 μM (Figure 7). Impressively, compound 86 demonstrated superior inhibitory effects on MCF-7 cells (IC_50_ = 6.64 μM) compared to cisplatin (IC_50_ = 10.23 μM) [80].

In 2019, five sesterterpenes, including 14,15-dehydro-6-*epi*-ophiobolin K (**91**), 14,15-dehydro-ophiobolin K (**92**), 14,15-dehydro-6-*epi*-ophiobolin G (**93**), 14,15-dehydro-ophiobolin G (**94**), and 14,15-dehydro-(*Z*)-14-ophiobolin G (**95**) were isolated from the alga-derived fungus *Aspergillus flocculosus* 168ST-16.1. The isolated compounds exhibited potent cytotoxicity against MDA-MB-231 cells with GI_50_ values ranging from 0.14 to 1.75 μM. Remarkably, compound **91** demonstrated inhibitory effects comparable to adriamycin (GI_50_ = 0.15 μM) [81]. The cytotoxicity differences might be attributed to the configuration of side chain double bonds (E-configuration optimizing binding) and the degree of unsaturation (tris-double bonds potentially reducing membrane permeability).

The marine sediment-derived fungus *Beauveria felina* KMM 4639 and the alga-derived fungus *Aspergillus carneus* KMM 4638, when co-cultured, produced three drimane-type sesquiterpenes asperflavinoids B (**96**), D (**97**), and E (**98**), which displayed cytotoxicity against MCF-7 cells with IC_50_ values of 54.1, 75.0, and 80.6 μM, respectively [82].

Three cycloheptapeptides, cordyheptapeptides C–E (**99**–**101**), were isolated from the fungus *Acremonium persicinum* SCSIO 115 derived from marine sediments. These peptides inhibited the proliferation of MCF-7 cells, with IC_50_ values of 3.0, 82.7, and 2.7 μM, respectively. Importantly, compounds **99** and **101** demonstrated significant cytotoxicity against MCF-7 cells, significantly surpassing the inhibitory effect of cisplatin (IC_50_ = 10.2 μM) [83].

A novel diketopiperazine compound, dichotocejpin A (**102**), was isolated from the deep-sea fungus *Dichotomomyces cejpii* FS110. This compound exhibited inhibitory activity against MCF-7 cells, with an IC_50_ value of 29.5 μM [84]. Compared with analogues containing disulfide bonds, dichotocejpin A showed relatively low cytotoxicity due to the absence of disulfide bonds in its molecular structure. However, the thio-methyl group in its molecule endowed the compound with excellent α-glucosidase inhibitory activity.

In 2018, the fungus *Trichothecium roseum* isolated from marine driftwood produced the cyclodipeptide trichomide D (**103**) in a salt environment. This compound exhibited significant cytotoxicity against MCF-7 cells, with an IC_50_ value of 0.079 μM, whereas cisplatin showed a much higher IC_50_ value of 19.440 μM against MCF-7 cells. Additionally, trichomide D, as a promising anticancer candidate, demonstrated inhibitory effects against SW480 and HL-60 cells superior to those of cisplatin, with IC_50_ values of 0.107 and 0.149 μM, respectively. The S configuration at the C-1γ position and the chlorohydrin structure were critical for its cytotoxicity [85,86].

Penithoketone (**104**), a naphthoquinone derivative, was isolated from the deep-sea fungus *Penicillium thomii* YPGA3, which inhibited the proliferation of MCF-7 and MDA-MB-468 cells (IC_50_ = 21 and 15 μM, respectively) [87].

A new cytochalasin, 19-hydroxycytochalasin B (**105**), was isolated from the fungus *Curvularia verruculosa* CS-129, which exhibited inhibitory activity against the proliferation of HCT-116, HepG-2, and MCF-7 cells with IC_50_ values ranging from 6.0 to 9.5 μM. Among them, **105** demonstrated the strongest inhibitory effect on MCF-7 cells (IC_50_ = 6.0 μM) [88]. In 2023, the fungus *Exophiala mesophila* produced three aranotin-type epipolythiodioxopiperazines, namely graphiumins K (**106**), L (**107**), and N (**108**), in a salt environment. These compounds exhibited cytotoxicity against MDA-MB-231 cells, with IC_50_ values of 3.7, 4.3, and 29.0 μM, respectively [89].

Among the 56 metabolites investigated, 13 demonstrated superior anti-breast cancer activity compared to clinically used drugs (Table 2). Importantly, the angucycline glycosides grincamycins B–F (**56**–**60**) exhibited potent inhibitory effects against the MCF-7 breast cancer cell line, outperforming both 5-fluorouracil (5-FU; IC_50_ = 35 μM) and doxorubicin (IC_50_ = 6.9 μM). Mechanistic studies revealed that grincamycin B targets isocitrate dehydrogenase 1, disrupting 2-oxoglutarate metabolism, redox balance, and inducing reactive oxygen species (ROS) accumulation, ultimately triggering apoptosis. Additionally, **56** was found to inhibit glioblastoma proliferation by targeting the RHOA and PI3K/AKT pathways, leading to G2/M cell-cycle arrest [90,91]. Another angucycline glycoside, marangucycline B (**61**), displayed 22-fold greater potency than cisplatin against MCF-7 cells, with structural analysis identifying the ketone group in its disaccharide moiety and the C-2″–O–C-3′ linkage as critical for its activity. Similarly, the oxo-anthraquinone kebanmycin A (**63**) exhibited six times stronger inhibition than adriamycin, with its C-7 hydroxyl group and naphthoxyanthraquinone skeleton playing key roles in cytotoxicity. Among cyclic peptides, bacillistatin 2 (**78**) demonstrated remarkable activity, surpassing valinomycin by threefold. Cordyheptapeptide E (**101**) and trichomide D (**103**) also exhibited potent cytotoxicity against MCF-7, with IC_50_ values of 2.7 μM and 0.079 μM, respectively, far exceeding those of cisplatin (IC_50_ = 10.2 μM and 19.44 μM). These findings highlight the therapeutic potential of microbial metabolites in breast cancer treatment. Their diverse mechanisms—ranging from metabolic disruption and ROS induction to cell-cycle arrest—provide a robust foundation for developing novel targeted therapies. Further exploration of their structure-activity relationships and synthetic accessibility could accelerate the discovery of next-generation anticancer agents.

### 3.3. Other Cancers

In addition to lung and breast cancer, colorectal cancer, prostate cancer, gastric cancer, and liver cancer represent major global health threats due to their high incidence and mortality rates. The limited efficacy and severe side effects of current treatments underscore the urgent need for novel anticancer agents [92,93,94]. Emerging research suggests that salt-adapted microbial metabolites produced by extremophilic microorganisms under high-salinity conditions hold significant promise in combating these malignancies.

**Figure 8:**
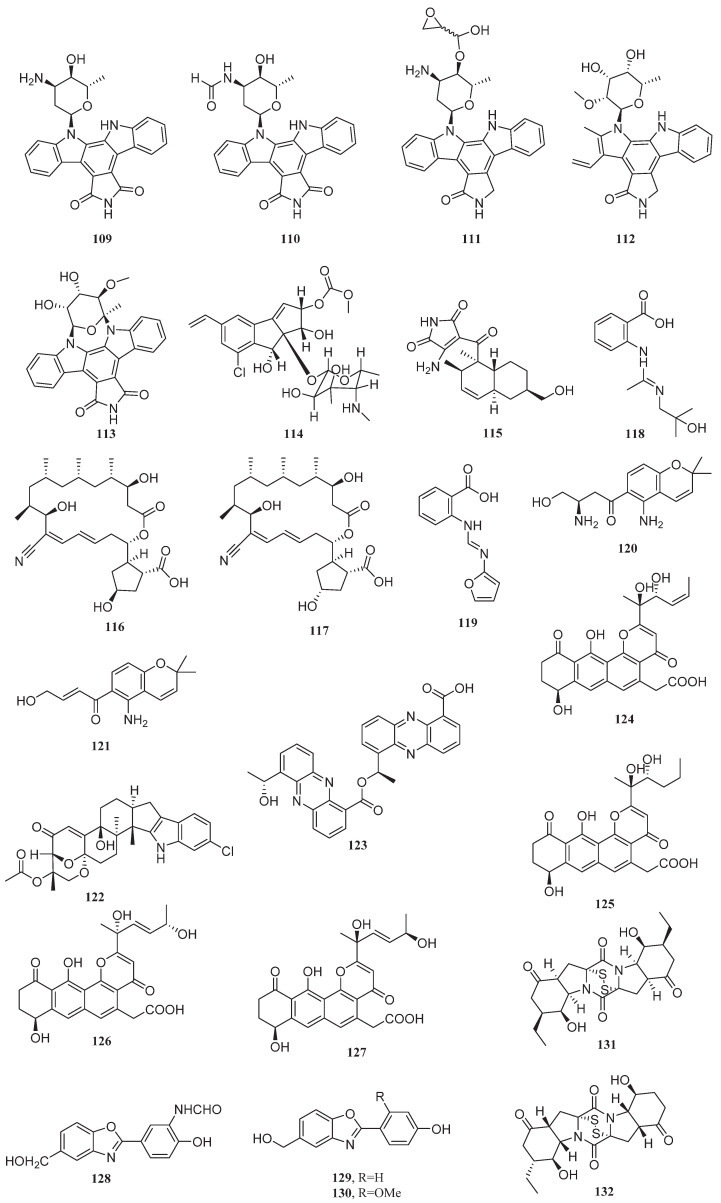
Chemical structures of compounds **109**–**132**.

For colon cancer, five staurosporine derivatives (Figure 8), including 7-oxo-holyrin A (**109**), 4′N-formyl-7-oxo-holyrin A (**110**), 3′-(hydroxyl(oxiran-2-yl)methoxy)-holyrine A (**111**), 3′-*epi*-5′-methoxy- K252d (**112**), and 7-oxo-MLR-52 (**113**), isolated from *Streptomyces* sp. NB-A13, exhibited IC_50_ values ranging from 0.16 to 9.54 μM against the human colon cancer cell line SW-620. These compounds showed stronger inhibitory effects on SW-620 compared with staurosporine (IC_50_ = 25.10 μM) [95]. The chlorinated polycyclic enediyne compound jejucarboside E (**114**), first isolated from the marine *Streptomyces* sp. JJC13, exhibited an IC_50_ value of 0.29 μM against the human colon cancer cell line HCT-116, demonstrating superior inhibitory effects compared with etoposide (IC_50_ = 0.56 μM). The presence of carbonate (-O-CO-O-) and methoxy (-OCH_3_) groups in its structure contribute to enhanced cytotoxicity [96]. When the halotolerant bacteria *Streptomyces* sp. GET02.ST and *Achromobacter* sp. GET02.AC, isolated from the cockroach gut, are co-cultured under 2.25% salt conditions, they produce the naphthalene compound ligiamycin B (**115**). Compound **115** exhibits cytotoxicity against HCT-116, with an IC_50_ value of 20.1 μM. The key to its cytotoxicity was closely associated with the structural modification of the C-16 hydroxyl (-OH) group [97]. The novel macrolides borrelidins C–D (**116**–**117**) were first isolated from *Nocardiopsis* sp. HYJ128, derived from saltern topsoil. These compounds exhibit IC_50_ values of 10 and 15 μM against HCT116, respectively. Borrelidin C (**116**) shows superior inhibitory efficacy against HCT116 compared to etoposide (14 μM) [98]. Anthranilic acid derivatives penipacid A (**118**) and penipacid E (**119**), isolated from the marine fungus *Penicillium paneum* SD-44, exhibited IC_50_ values of 8.4 and 9.7 μM, respectively, against the human colon cancer cell line RKO [99]. The halotolerant fungus *Fusarium equiseti* UBOCC-A-117302 produces the fusarochromanone derivatives deacetylfusarochromene (**120**) and deacetamidofusarochrom-2′,3-diene (**121**), which exhibit inhibitory activity against HCT-116 cells with EC_50_ values of 0.087 and 13.730 μM, respectively, superior to that of staurosporine [100].

Alkaloid asperindole A (**122**), derived from the endophytic fungus *Aspergillus candidus* KMM 4676, exhibits inhibitory effects by inducing S-phase arrest in the human prostate cancer cell line 22Rv1 (IC_50_ = 4.86 μM). Homolog comparison indicated that the key cytotoxic structure was the acetoxy group at C-27, and its activity significantly decreased when this group was substituted [101]. Phenazine compound phenazostatin J (**123**) produced by *Cystobasidium laryngis* IV17-028 exhibited an IC_50_ value of 0.0077 μM against the human gastric cancer cell line NUGC-3, displaying 19.5-fold stronger activity compared to adriamycin (IC_50_ = 0.1500 μM). The cytotoxicity of the compound may be associated with the hydroxyethyl group and the ester linkage in its structure [102].

Among anti-hepatocellular carcinoma metabolites, the pyrone compound shellmycins A-D (**124**–**127**), first isolated from *Streptomyces* sp. shell-016, exhibited proliferation inhibitory activity against human hepatocellular carcinoma cell line HepG2 (IC_50_ = 4.22, 5.67, 11.30, and 5.16 μM, respectively), displaying enhanced activity compared to cisplatin (IC_50_ > 50 μM) [103]. *Nocardiopsis lucentensis* DSM 44048, isolated from salt marsh soil, produces benzoxazole derivatives nocarbenzoxazole D (**128**), nocarbenzoxazole F (**129**), and nocarbenzoxazole G (**130**). These compounds exhibit inhibitory effects against HepG2 cells, with IC_50_ values of 47, 16, and 3 μM, respectively [104]. Polyketide compound rostratin C (**131**) secreted by marine *Epicoccum nigrum* SD-388 exhibited an IC_50_ value of 4.88 μM against the human hepatocellular carcinoma cell line Huh7.5, displaying superior inhibitory activity compared to the clinical first-line anti-hepatocellular carcinoma drug sorafenib (IC_50_ = 8.2 μM). The disulfide bridge at positions C-2/C-2′ may be critical for its cytotoxicity. From the same fungal source, 7′-demethoxyrostratin C (**132**) also exhibited cytotoxicity against Huh7.5, with an IC_50_ value of 9.52 μM. Notably, rostratin C was first isolated from the marine fungus *Exserohilum rostratum* CNK-630, also demonstrating inhibitory effects against HCT-116 (IC_50_ = 0.76 μg/mL) [105,106].

This section highlights 15 bioactive metabolites from halophilic and halotolerant microorganisms, showcasing potent cytotoxicity against diverse cancer types (Table 3). These compounds, produced by extremophiles adapted to high-salinity environments, often feature unique pharmacophores that differentiate them from conventional therapeutics. In vitro and in vivo studies demonstrate their selective anticancer activity, suggesting interactions with critical oncogenic pathways. However, further research is required to fully elucidate their molecular mechanisms and therapeutic potential.

## 4. Perspectives

### 4.1. Uniqueness of Metabolites from Salt-Adapted Microorganisms and Their Environmental Association

Salt-adapted microorganisms evolved unique metabolic strategies under high-salt stress, and their secondary metabolites exhibited dual characteristics of structural novelty and environmental dependency. For example, *Streptomyces* sp. IMB094 produced neo-actinomycins A-B (**4**–**5**) with a unique tetracyclic chromophore in salt-containing environments, while only generating conventional tricyclic actinomycins consistent with ordinary microorganisms in non-salt conditions, highlighting the decisive regulatory role of salt ions in metabolic pathways. The marine fungus *Penicillium terrestre* synthesized terrestrol A (**29**), the first gentisyl alcohol trimer in nature, under high-salt conditions. *Streptomyces* sp. S063 generated cyclic peptides lenziamide D1 and B1 (**6**–**7**) in a 3% salt environment, which were the first cyclic decapeptides containing Piz with negative chemical shifts. Their five-site N-methylation pattern and atypical amino acid combinations were extremely rare in natural peptides. Kebanmycin A (**63**), isolated from mangrove-halotolerant actinomycetes, had a 6/6/6/6/6/6 pyrano-naphtho-xanthene hexacyclic skeleton, lacking a C-7 hydroxyl group and with a glycosyl O-methylation modification, representing a rare new structure of polycyclic flavonoids in natural products. 10-*epi*-HSAF (**68**) from deep-sea streptomycetes, as the enol-form 10-*epi* isomer of HSAF, had a 5S absolute configuration and enol structure that were extremely rare in nature. The presence of salt ions (e.g., Cl^−^/Br^−^) in salt environments significantly promoted the synthesis of halogenated metabolites: *Aspergillus* sp. SCSIO F063 induced brominated anthraquinone formation upon NaBr addition and promoted chloride synthesis with sea salt; *Streptomyces* sp. OUCMDZ-1703 utilized high-chlorine environments to synthesize chlorinated polyketides strepchloritides A−B (**66**–**67**); *Spicaria elegans* KLA-03 produced the chlorinated compound trichodermamide B only under high-salt conditions; and *Aspergillus variecolor* B-17 synthesized chlorinated derivatives from chlorine in the medium [107,108]. These metabolites were synthesized via salt ion-induced specific enzyme catalysis and low-homology gene cluster pathways. Their unconventional skeletons, specific functional group modifications, and stereochemical diversity not only served as unique chemical markers of salt-adapted microorganisms in extreme environments but also provided irreplaceable structural templates for innovative antitumor drug development. They were expected to reduce drug resistance and side effects of traditional chemotherapeutics by targeting specific tumor cell markers.

### 4.2. Diversity of Salt-Adapted Microorganisms

**Figure 9:**
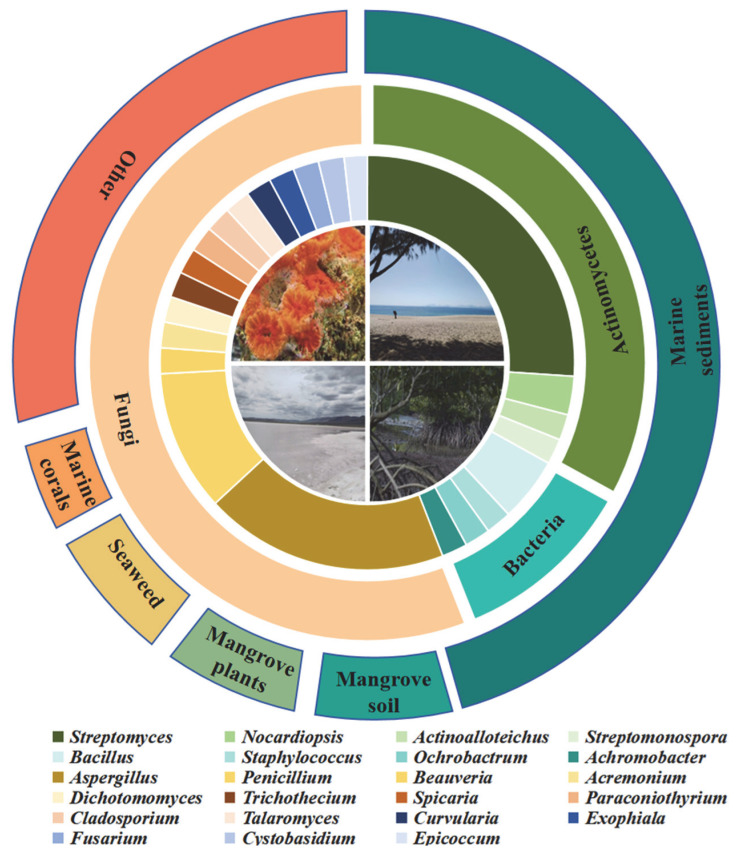
Diversity of Salt-Adapted Microorganisms.

The microorganisms collected in this review exhibited rich diversity, encompassing bacteria and fungi, with Actinobacteria and Ascomycota dominating. At the generic level, Streptomyces, Aspergillus, and Penicillium were core taxa producing cytotoxic metabolites (as shown in Figure 9). The ecological distribution of these microorganisms was highly dependent on marine-related habitats—marine sediments served as the primary aggregation site due to their rich nutrients, small temperature fluctuations, and abundant microbial attachment substrates. Additionally, the microbial communities in mangrove ecosystems also demonstrated remarkable species abundance and metabolic diversity.

### 4.3. Research Gaps and Challenges

Current research on salt-adapted microorganisms in anticancer drug development still faces numerous gaps and challenges. Most studies only evaluated activity through in vitro cytotoxicity assays such as MTT and SRB, lacking a systematic analysis of cytotoxic mechanisms. As the vast majority of active compounds had not undergone target validation, their action pathways and structure-activity relationships could not be elucidated, severely restricting the transformation of related metabolites from basic research to innovative drugs. Except for marine environments, microbial resources in extremely high-salt environments (with salt concentrations up to saturation), such as salt lakes, saline-alkali soils, and salt mines, showed significant gaps [109]. Secondary metabolites produced in these extreme environments might possess novel chemical structures and biological activities, but were constrained by the technical bottlenecks of traditional pure culture methods. Difficulties in reproducing in-situ nutritional conditions, simulating microbial community symbiotic metabolic interaction networks, and meeting extreme physiological requirements resulted in the failure to successfully culture numerous microorganisms with phylogenetic uniqueness, novel metabolic pathways, and key ecological functions [110,111,112]. Inadequate elucidation of biosynthetic mechanisms, including the poorly analyzed coupled pathways of compatible solute synthesis and secondary metabolism in salt-adapted microorganisms, as well as the unclarified regulatory mechanisms of key enzyme activities under high-salt conditions, further restricts the transformation efficiency from laboratory discovery to industrial application [113,114,115].

## 5. Conclusions

Salt-adapted microorganisms represent a promising yet underexplored source of novel anticancer agents. Over the past two decades, studies have identified bioactive metabolites from these extremophiles with potent activity against major malignancies, including lung and breast carcinomas. While these discoveries open new avenues for drug development, clinical translation faces significant hurdles. Current research remains largely confined to in vitro models, underscoring the need for deeper investigation into pharmacokinetics, safety profiles, and therapeutic efficacy. Furthermore, the biosynthetic pathways and exact mechanisms of cytotoxicity of these compounds remain elusive, posing challenges for scalable production and structural optimization. Nevertheless, emerging technologies, such as multi-omics, synthetic biology, and precision fermentation, are poised to overcome these limitations. By leveraging these tools, researchers can accelerate the development of halophile-derived anticancer therapies, bridging the gap between microbial discovery and clinical application. Advancements in genomics and metabolomics are set to revolutionize the discovery of anticancer agents from halophilic and halotolerant microorganisms. High-throughput screening technologies will enable systematic identification of previously unknown bioactive molecules, not only expanding the pipeline of novel drug candidates but also diversifying the microbial resource pool for anticancer research. These developments promise to sustain long-term innovation in oncology drug development. In summary, halophilic and halotolerant microorganisms remain an underexplored yet highly promising frontier in anticancer drug discovery. As research progresses, the translation of these microbial natural products into clinical applications may yield transformative breakthroughs in cancer therapy.

## Figures and Tables

**Table 1:** Secondary metabolites produced by salt-adapted microorganisms with significant cytotoxicity against lung cancer cell lines.

No	Metabolites	Classes	Microorganisms	The Tested Tumor Cells IC_50_ Values	Positive Control IC_50_ Values	Ref
**19**	21-*epi*-Ophiobolin O	Ophiobolin	*Aspergillus ustus* 094102	A549 0.6 μM	Etoposide 0.63 μM	[50]
**22**	Nigerapyrone E	α-pyrone derivative	*Aspergillus niger* MA-132	A549 43 μM	Fluorouracil 52 μM	[52]
**39**	Sumalarin A	Sulfur-containing curvularin derivative	*Penicillium sumatrense* MA-92	NCI-H460 3.8 μM	5-fluorouracil 8.5 μM	[57]
**40**	Sumalarin B	Sulfur-containing curvularin derivative	*Penicillium sumatrense* MA-92	NCI-H460 4.6 μM	5-fluorouracil 8.5 μM	[57]
**41**	Sumalarin C	Sulfur-containing curvularin derivative	*Penicillium sumatrense* MA-92	NCI-H460 7.0 μM	5-fluorouracil 8.5 μM	[57]
**42**	Brocazine A	Diketopiperazine derivative	*Penicillium brocae* MA-231	NCI-H460 4.9 μM	Cefitinib 7.6 μM	[58]
**43**	Brocazine B	Diketopiperazine derivative	*Penicillium brocae* MA-231	NCI-H460 4.0 μM	Cefitinib 7.6 μM	[58]
**44**	Brocazine F	Diketopiperazine derivative	*Penicillium brocae* MA-231	NCI-H460 0.89 μM	Cefitinib 7.6 μM	[58]
**54**	(*R*)-6-((8*R*)-hydroxypropyl)-2-methyl-5,6-dihydro- 4H-pyran-4-one	Pyranone	*Cladosporium halotolerans* FS702	A-549 0.23 μM	Doxorubicin 1.38 μM	[64]

**Table 2:** Secondary metabolites produced by salt-adapted microorganisms with significant cytotoxicity against breast cancer cell lines.

No	Metabolites	Classes	Microorganisms	The Tested Tumor Cells IC_50_ Values	Positive Control IC_50_ Values	Ref
**56**	Grincamycin B	C-glycoside angucyclines	*Streptomyces lusitanus* SCSIO LR32	MCF-7 12 μM	5-Fluorouracil/ doxorubicin 35/6.9 μM	[66]
**57**	Grincamycin C	C-glycoside angucyclines	*Streptomyces lusitanus* SCSIO LR32	MCF-7 11 μM	5-Fluorouracil/ doxorubicin 35/6.9 μM	[66]
**58**	Grincamycin D	C-glycoside angucyclines	*Streptomyces lusitanus* SCSIO LR32	MCF-7 6.1 μM	5-Fluorouracil/ doxorubicin 35/6.9 μM	[66]
**59**	Grincamycin E	C-glycoside angucyclines	*Streptomyces lusitanus* SCSIO LR32	MCF-7 8.7 μM	5-Fluorouracil/ doxorubicin 35/6.9 μM	[66]
**60**	Grincamycin F	C-glycoside angucyclines	*Streptomyces lusitanus* SCSIO LR32	MCF-7 19 μM	5-Fluorouracil/ doxorubicin 35/6.9 μM	[66]
**61**	Marangucycline B	C-glycoside angucycline	*Streptomycetes* sp. SCSIO 11594	MCF-7 0.24 μM	Cisplatin 5.26 μM	[67]
**63**	Kebanmycin A	Polycyclic xanthones	*Streptomyces* sp. SCSIO 40068	MCF-7 0.12 μM	Adriamycin 0.72 μM	[69]
**68**	10-*epi*-HSAF	Polycyclic tetramate macrolactam	*Streptomyces* sp. SCSIO 40010	MCF-7 2.47 μM	Cisplatin 3.19 μM	[71]
**78**	Bacillistatin 2	Cyclodepsipeptide	*Bacillus silvestris*	MCF-7 0.00031 μg/mL	Valinomycin 0.00100 μg/mL	[77]
**86**	(1′*S*)-6-O-methyl-7-chloroaverantin	Chlorinated anthraquinone	*Aspergillus* sp. SCSIO F063	MCF-7 6.64 μM	Cisplatin 10.23 μM	[80]
**91**	14,15-dehydro-6-*epi*-ophiobolin K	Sesterterpene	*Aspergillus flocculosus* 168ST-16.1	MDA-MB-231 0.14 μM	Adriamycin 0.15 μM	[81]
**101**	Cordyheptapeptide E	Cycloheptapeptide	*Acremonium persicinum* SCSIO 115	MCF-7 2.7 μM	Cisplatin 10.2 μM	[83]
**103**	Trichomide D	Cyclodepsipeptide	*Trichothecium roseum*	MCF-7 0.079 μM	Cisplatin 19.44 ± 1.57 μM	[85]

**Table 3:** Secondary metabolites produced by salt-adapted microorganisms with significant cytotoxicity against other cancer cell lines.

No	Metabolites	Classes	Microorganisms	The Tested Tumor Cells IC_50_ Values	Positive Control IC_50_ Values	Ref
**109**	7-oxo-holyrin A	Staurosporine derivative	*Streptomyces* sp. NB-A13	SW-620 2.14 μM	Staurosporine 25.1 μM	[95]
**110**	4′N-formyl-7-oxo-holyrin A	Staurosporine derivative	*Streptomyces* sp. NB-A13	SW-620 0.74 μM	Staurosporine 25.1 μM	[95]
**111**	3′-(hydroxyl(oxiran-2-yl)methoxy)-holyrine A	Staurosporine derivative	*Streptomyces* sp. NB-A13	SW-620 2.00 μM	Staurosporine 25.1 μM	[95]
**112**	3′-*epi*-5′-methoxy-K252d	Staurosporine derivative	*Streptomyces* sp. NB-A13	SW-620 9.54 μM	Staurosporine 25.10 μM	[95]
**113**	7-oxo-MLR-52	Staurosporine derivative	*Streptomyces* sp. NB-A13	SW-620 0.16 μM	Staurosporine 25.10 μM	[95]
**114**	Jejucarboside E	Chlorinated polycyclic enediyne	Streptomyces sp. JJC13	HCT 116 0.29 μM	Etoposide 0.56 μM	[96]
**116**	Borrelidin C	Macrolide	*Nocardiopsis* sp. strain HYJ128	HCT116 10 μM	Etoposide 14 μM	[98]
**120**	Deacetylfusarochromene	Fusarochromanone derivative	*Fusarium equiseti* UBOCC-A-117302	HCT-116 0.087 μM	Staurosporine 25.7 μM	[100]
**121**	Deacetamidofusarochrom-2′,3-diene	Fusarochromanone derivative	*Fusarium equiseti* UBOCC-A-117302	HCT-116 13.730 μM	Staurosporine 25.7 μM	[100]
**123**	Phenazostatin J	Phenazine	*Cystobasidium laryngis* IV17-028	NUGC-3 0.0077 μM	Adriamycin 0.15 μM	[102]
**124**	Shellmycin A	Pyrone	*Streptomyces* sp. shell-016	HepG-2 4.22 µM	Cisplatin >50 µM	[103]
**125**	Shellmycin B	Pyrone	*Streptomyces* sp. shell-016	HepG-2 5.67 µM	Cisplatin >50 µM	[103]
**126**	Shellmycin C	Pyrone	*Streptomyces* sp. shell-016	HepG-2 11.30 µM	Cisplatin >50 µM	[103]
**127**	shellmycin D	Pyrone	*Streptomyces* sp. shell-016	HepG-2 5.16 µM	Cisplatin >50 µM	[103]
**131**	Rostratin C	Polyketide	*Epicoccum nigrum* SD-388	Huh7.5 4.88 μM	Sorafenib 8.2 μM	[106]

## Data Availability

No new data were created or analyzed in this study. Data sharing is not applicable to this article.
